# Effect of silicone oil removal timing on functional and structural outcomes in primary rhegmatogenous retinal detachment

**DOI:** 10.1186/s40942-026-00809-2

**Published:** 2026-02-19

**Authors:** Ahmed Mohamed Youssef Moussa, Ahmed Saad Albalkini, Hisham Ashraf Omar, Tamer A. Macky, Ayman Khattab, Abdussalam Mohsen Abdullatif

**Affiliations:** Department of Ophthalmology, Kasr AlAiny Hospital, Cairo University, 9th, El Henedi Street, Apt. # 3, Haram, Giza, Cairo 12111 Egypt

**Keywords:** Rhegmatogenous retinal detachment, Silicone oil removal, Microperimetry, OCTA, Visual outcomes, Tamponade duration.

## Abstract

**Purpose:**

To compare anatomical and functional outcomes following early (2 months) versus delayed (6 months) silicone oil (SO) removal after pars plana vitrectomy (PPV) for primary rhegmatogenous retinal detachment (RRD).

**Methods:**

This prospective, randomized, single-center study included 66 eyes with primary macula off RRD and proliferative vitreoretinopathy grade ≤ B. Patients were randomized into two groups: Group A (early SO removal, 2 months) and Group B (delayed SO removal, 6 months). All surgeries were standardized, and postoperative evaluations included corrected distance visual acuity (CDVA), microperimetry, and optical coherence tomography angiography (OCTA) one month after SO removal. Primary outcomes were functional (CDVA, retinal sensitivity) and anatomical (reattachment rate, OCTA parameters) comparisons between groups.

**Results:**

At one month post-SO removal, both groups achieved similar primary and final retinal reattachment rates (*P* = 0.237). Mean CDVA did not differ significantly between groups (0.83 ± 0.26 vs. 0.91 ± 0.29 logMAR, *P* = 0.147). Microperimetry showed significantly higher retinal sensitivities in Group A across the outer (*P* < 0.001), middle (*P* = 0.013), and overall macular areas (*P* = 0.029). OCTA parameters, including vessel densities and choroidal flow area, were comparable (all *P* > 0.05). No cases of silicone oil–related visual loss were observed, and postoperative complications were infrequent and similar between groups.

**Conclusions:**

Early SO removal resulted in superior microperimetric retinal sensitivity without compromising anatomical success or increasing postoperative complications. Although CDVA was similar, microperimetry provided additional insight into visual function, underscoring its value in postoperative assessment following PPV for RRD.

## Introduction

Intraocular gas is generally preferred for less severe rhegmatogenous retinal detachment (RRD) while silicone oil (SO) tamponade is reserved for more complex cases such as established or high risk of proliferative vitreoretinopathy (PVR), giant tears, uveitis, or choroidal detachment [1]. Although the safety and efficacy of SO in ophthalmic surgery has been well established, concerns regarding its potential retinal toxicity have received attention. Accordingly, the optimal timing of SO removal remains controversial. Recent studies directly comparing early versus delayed SO removal have reported conflicting outcomes. Early removal (≤ 3 months) has been associated with fewer SO-related complications, including emulsification, secondary glaucoma, and keratopathy, without significantly compromising anatomical or visual outcomes in selected patients [2, 3]. In contrast, other reports suggest that shorter tamponade duration may be associated with a trend toward higher retinal redetachment rates, particularly in high-risk eyes, although this association is not consistently statistically significant [4].

Additionally, vision loss after SO removal may occur due to cystoid macular oedema (CMO), hypotony, optic neuropathy, or epiretinal membranes, but some cases remain unexplained and are termed SO-related visual loss (SORVL) [5]. SORVL, defined as loss of > 2 Snellen lines during or after tamponade without other cause, has been observed particularly in giant retinal tears and macular-sparing RRD, with incidence up to 50% [6]. Proposed mechanisms include tamponade duration, altered retinal metabolism, increased growth factors, phototoxicity, and depletion of antioxidants and lipid-soluble factors [6]. Clinical findings may involve inner retinal thinning, scotoma, field defects, and impaired electrophysiology, with some evidence of impaired microcirculation [3]. Furthermore the retinal vascular density and perfusion density in the superficial capillary plexus gradually decreased over time [7]. 

Owing to these competing advantages and disadvantages, there is no consensus regarding the ideal timing of SO removal, highlighting the need for further evaluation. To address these concerns, our study aims to compare early SO removal at 2 months versus delayed removal at 6 months on anatomical and functional outcomes in primary macula off RRD.

## Patients and methods

This prospective, single-center, randomized, interventional comparative study was conducted at Kasr Al Aini Hospital, Cairo University, between December 2022 and December 2024. Patients with primary macula off rhegmatogenous retinal detachment (RRD) were enrolled after providing informed consent. The study protocol was approved by the Research Ethics Committee (REC) of Cairo University and adhered to the tenets of the Declaration of Helsinki.

### Study population

Inclusion criteria were patients ≥ 18 years with primary macula off RRD and proliferative vitreoretinopathy (PVR) up to grade B, according to the updated Retina Society classification of Machemer at al., in 1991 [8]. Exclusion criteria included previous RD surgery, open globe injuries, pre-existing macular or vascular retinal pathologies (e.g., diabetic retinopathy, vein occlusions), primary surgical failure (recurrent detachment under SO), postoperative refractory glaucoma due to early emulsification, postoperative CDVA < CF at 1 m, or eyes that developed macular hole or epiretinal membrane.

Eligible patients were simply randomized by simple computer-generated numbers (Microsoft Excel©) into:

- **Group A**: SO removal at 2 months after primary repair.
- **Group B**: SO removal at 6 months after primary repair.

### Preoperative evaluation

Data collected included age, sex, and ocular/systemic history. Ophthalmic examination included CDVA (Snellen, converted to logMAR), slit-lamp for lens status, intraocular pressure (IOP) by Goldmann applanation, and fundus evaluation (indirect ophthalmoscopy and slit-lamp biomicroscopy) for extent of RRD, macular status, PVR grade, and number of breaks. Axial length was also measured using A scan.

### Surgical technique

All surgeries were done under local anesthesia. Combined phacoemulsification PPV depends on the degree of cataract. If the cataract interferes with visualization combined surgery was done, but if the lens is clear or cataractous but doesn’t interfere with visualization, the lens was kept and removed later with silicone removal. 23-gauge vitrectomy was done using noncontact wide-angle viewing system and Constellation vitrectomy machine (Alcon Laboratories, Inc., Fort Worth, TX, USA). Posterior hyaloid detachment was peeled up to the posterior border of vitreous base or up to line where it is strongly adherent. Any epiretinal membranes/tissues were removed but ILM was not removed. Central retina was stabilized by perfluorocarbon liquid (PFCL) which was injected gently up to the posterior side of the retinal break. Then, through existing retinal breaks or peripheral retinotomy, when necessary, subretinal fluid (SRF) was drained. Under scleral indentation, complete vitreous base shaving was performed. After complete retinal reattachment was achieved, endo-laser photocoagulation was applied 360-degree and to any retinal breaks, followed by air/fluid exchange. Silicone oil (SO) (5,000 centistokes) was injected. Sclerotomies were sutured, when needed, with Vicryl 8.0 sutures.

### Postoperative evaluation

Patients were examined at Day 1, Week 1, and 1 month post-SO removal. Each visit included CDVA, IOP, and fundus examination for retinal status.

At 1 month post-SO removal, macular sensitivity was assessed using microperimetry. Microperimetry was performed using the MP-3 microperimeter (NIDEK Co., Ltd., Gamagori, Japan) under mesopic conditions. Testing was conducted in a dimly lit room after correcting refractive error for the testing distance. Microperimetry outcomes were analyzed using ring-based analysis centered on the fovea. Retinal sensitivity values were averaged within concentric rings corresponding to predefined eccentricities from the foveal center (e.g., 0–2°, 2–4°, and 4–6°). Mean sensitivity for each ring was calculated in decibels (dB).

Also, at 1 month post SO removal, macular circulation was evaluated using 6 × 6 mm optical coherence tomography angiography (OCTA) imaging (RTVue XR Avanti, AngioVue; Optovue Inc., Fremont, CA, USA). All vessel density parameters were obtained using the built-in analysis software of the OCTA device. Vessel density was defined as the proportion of the analyzed area occupied by perfused vasculature and was automatically calculated by the device software following proprietary motion correction, segmentation, and binarization algorithms. Analyses were performed on predefined retinal layers whether superficial or deep capillary plexuses and within standardized regions (foveal, parafoveal) as provided by the device software. Images with low signal strength or significant motion artifacts were excluded according to predefined quality criteria.

Both microperimetry and OCTA measurements were obtained by trained examiner who was masked to clinical information and study group allocation at the time of image acquisition and analysis.

### Outcome measures

***Primary outcomes*** at 1 month post-SO removal is to compare the following parameters between the two study groups:

- Functional parameters: CDVA and macular sensitivity (microperimetry).
- Anatomical parameters: primary and final retinal reattachment rate and macular microcirculation/structural changes (OCTA).

***Secondary outcomes*** include comparison of postoperative complications (macular hole, epiretinal membrane, and cystoid macular oedema) between the two study groups.

### Sample size calculation and statistical analysis

The sample size was calculated based on Zhou et al. (2020) [9] to detect a 3% difference in macular vascular density (SD = 4%) between two groups of RRD with 80% power and α = 0.05, yielding 62 eyes (31 per group). Allowing for a 10% dropout, the final sample size was 68 eyes.

Data were revised, coded, and analyzed using SPSS version 25 (IBM Corp., Armonk, NY). Normality was tested with the Shapiro–Wilk test. Descriptive statistics were expressed as mean ± SD, median and range for numerical data, and frequency and percentage for categorical data. Comparisons between groups were performed using the Student’s t-test or Mann–Whitney U test as appropriate, while associations between categorical variables were assessed using the Chi-square or Fisher’s exact test. Correlations between quantitative variables were evaluated, and diagnostic performance was analyzed using ROC curves with AUC interpretation. A *P*-value *<* 0.05 was considered statistically significant.

## Results

Sixty-eight eyes of 68 patients were operated. Two patients didn’t show up during follow up in group B and were excluded from the study, so 66 eyes of 66 patients were included in this study; 34 in group A and 32 in group B with their demographics and baseline features summarized in Table 1. All pars plana vitrectomy surgeries were performed by the same surgeon and all silicone oil removal surgeries were performed by another but same surgeon.

**Table 1:** Baseline features

	Group A *n* = 34	Group B *n* = 32	*P* value
Eye, OD *n* (%)	14 (41.2%)	19 (59.4%)	0.31
Age, years, mean ± SD	48.75 ± 11.71	49.07 ± 15.23	0.927
Sex, male n (%)	26 (76.5%)	20 (62.5%)	0.271
RD duration, mean ± SD			
Lens status, n (%)			
Phakic Pseudophakic	11 (32.4%) 23 (67.6%)	10 (31.4%) 22 (68.6%)	0.307
No. of breaks, n (%)	1.38 ± 0.55	1.40 ± 0.81	0.618
Baseline CDVA, logMAR, mean ± SD	1.70 ± 0.38	1.71 ± 0.43	0.114
Axial length (mm), mean ± SD	26.25 ± 1.81	26.67 ± 2.19	0.416

Thirty eyes (88.2%) in Group A, and 23 eyes (71.9%) in Group B underwent pars plana vitrectomy (PPV) without combined phacoemulsification (*P* = 0.132). Among the phakic eyes, 4 of 11 eyes (36.4%) in Group A and 9 of 10 eyes (90%) in Group B underwent combined phacoemulsification with intraocular lens (IOL) implantation and 3-port PPV (*P* = 0.03). The remaining phakic eyes—7 in Group A and 1 in Group B—underwent phacoemulsification at the time of silicone oil removal.

### Postoperative data

#### Anatomical outcomes

At one-month post silicone oil removal, there was no statistically significant difference between both groups in primary or final anatomical success (*P* = 0.237) (Table 2). Three cases of postoperative redetachment occurred shortly after SO removal within the first month and were operated once discovered while the MH case was discovered at one month but was followed up and operated at the 3rd month. Accordingly, all those cases were excluded from the OCTA analysis.

**Table 2:** Outcomes of both groups at one-month post silicone oil removal

	Group A *n* = 34	Group B *n* = 32	*P* value
Primary anatomical success, *n* (%)	31 (91.17%)	32 (100%)	0.237
Final anatomical success, n (%)	34 (100%)	32 (100%)	
Secondary intervention, n (%)			
For reattachment For Macular Hole repair	3 (8.8%)	1 (3%)	0.237
CDVA, logMAR, mean ± SD	0.83 ± 0.26	0.91 ± 0.29	0.147
IOP, mmHg, mean ± SD	12.19 ± 3.02	11.43 ± 2.36	0.28

#### Optical coherence tomography angiography (OCTA)

There was no statistically significant difference between both groups in central foveal thickness (CFT) (*P* = 0.684) or any other OCTA parameter. The choroidal flow area (*P* = 0.704), FAZ area (*P* = 0.268), and FAZ perimeter (*P* = 0.065) were comparable. Superficial and deep vascular plexus vessel densities showed no statistically significant difference across all regions, with all *P*-values > 0.05 (Table 3).

**Table 3:** OCTA outcomes of both groups at one-month post silicone oil removal

	Group A (*n* = 31) *	Group B (*n* = 31) *	*P* value
CFT (µm), mean ± SD	266.56 ± 44.72	261.80 ± 46.97	0.684
Choroidal flow area (mm^2^), mean ± SD	2.05 ± 0.39	2.00 ± 0.33	0.704
FAZ area (mm^2^), mean ± SD perimeter (mm), mean ± SD	0.41 ± 0.68 2.45 ± 1.01	0.26 ± 0.09 2.03 ± 0.48	0.268 0.065
SVP vessel density (%), mean ± SD			
Fovea Parafovea Temporal Parafovea Nasal Parafovea Superior Parafovea Inferior Parafovea Perifovea	21.88 ± 12.07 39.40 ± 8.48 38.91 ± 8.06 38.09 ± 9.85 39.50 ± 11.14 40.57 ± 9.66 41.30 ± 7.51	25.46 ± 9.26 37.07 ± 6.91 37.68 ± 6.89 35.45 ± 9.37 38.02 ± 7.93 37.36 ± 7.22 38.71 ± 4.88	0.198 0.242 0.524 0.285 0.390 0.147 0.114
DVP vessel density (%), mean ± SD			
Overall DVP Fovea Parafovea Temporal Parafovea Nasal Parafovea Superior Parafovea Inferior Parafovea Perifovea	40.32 ± 7.89 30.49 ± 13.35 43.20 ± 11.60 44.57 ± 12.77 44.28 ± 10.50 43.66 ± 12.64 42.04 ± 13.18 40.57 ± 7.46	41.26 ± 6.56 34.57 ± 12.74 44.76 ± 8.40 44.89 ± 10.88 45.25 ± 8.13 44.84 ± 10.34 44.44 ± 7.98 41.57 ± 7.96	0.597 0.223 0.811 0.698 0.685 0.762 0.751 0.378

*Analysis was performed on all eyes, excluding the three eyes with recurrent retinal detachment in group A and the single eye in group B that developed a macular hole

### Functional outcomes

#### Corrected distance visual acuity (CDVA)

At one month following silicone oil removal, there was no statistically significant difference in mean CDVA between the two groups among successfully reattached eyes (63 eyes; 31 in Group A and 32 in Group B). The mean CDVA (logMAR) was 0.83 ± 0.26 in Group A and 0.91 ± 0.29 in Group B (*P* = 0.147) **(**Table 2**)**.

**Fig. 1:**
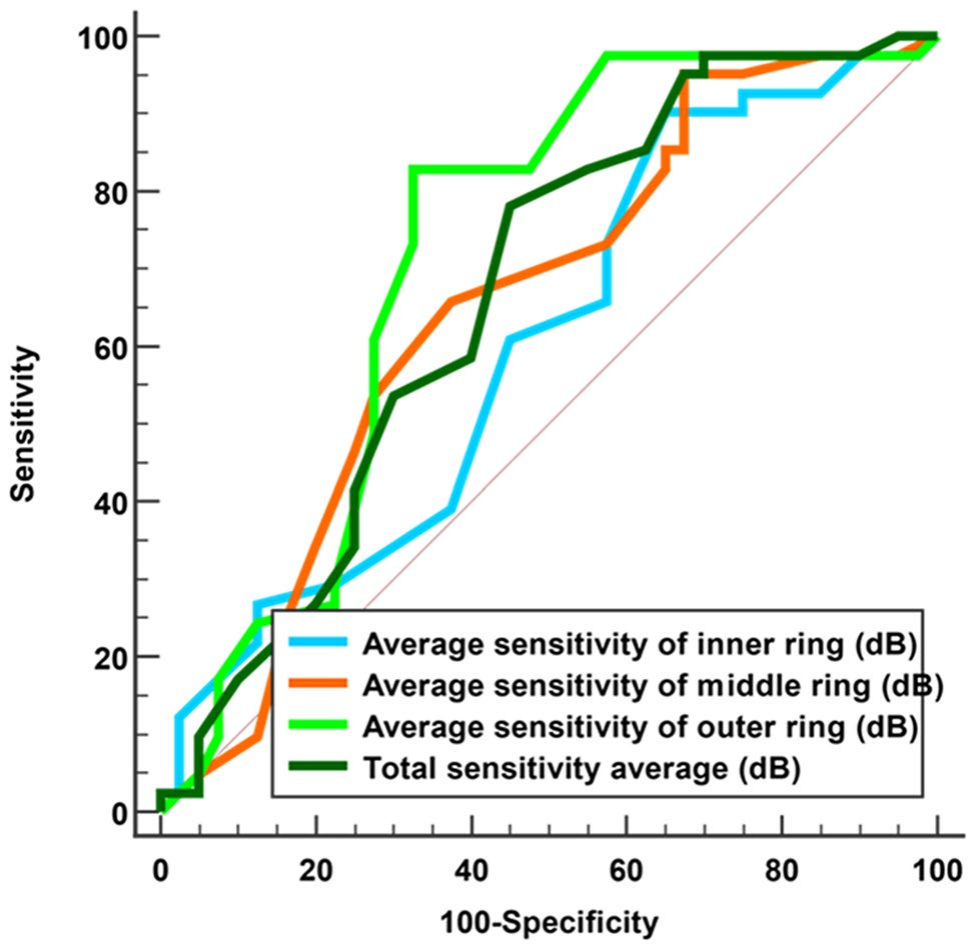
ROC curves of retinal sensitivity parameters for discrimination between study groups

**Microperimetry** (Analysis was performed on all eyes, excluding the three eyes with recurrent retinal detachment in Group A and the single eye in Group B that developed a macular hole). At one month after silicone oil removal, Group A showed significantly higher retinal sensitivity than Group B in the outer ring (18.53 ± 4.42 vs. 14.47 ± 6.27 dB; *P* < 0.001), middle ring (20.84 ± 4.27 vs. 18.07 ± 5.77 dB; *P* = 0.013), and overall macular areas (19.31 ± 4.35 vs. 16.43 ± 5.71 dB; *P* = 0.029), while the inner ring difference was not significant (*P* = 0.122) (Table 4). The outer ring demonstrated the strongest diagnostic accuracy (AUC = 0.722), followed by the overall macular area (AUC = 0.666) and middle ring sensitivities (AUC = 0.650), with the inner ring showing the weakest accuracy (AUC = 0.603) **(**Fig. 1**)**.

**Table 4:** Microperimetry results showing retinal sensitivity in outer, middle, inner ring in both groups

	Group A	Group B	Test, *p*- value
	*n* = 31	*n* = 31	
Average sensitivity of outer ring (dB), Mean ± SD	18.53 ± 4.42	14.47 ± 6.27	*p* < 0.001
Average sensitivity of middle ring (dB), Mean ± SD	20.84 ± 4.27	18.07 ± 5.77	*p* = 0.034
Average sensitivity of inner ring (dB), Mean ± SD	18.09 ± 5.29	15.87 ± 5.89	*p* = 0.122
Total sensitivity average (dB), Mean ± SD	19.31 ± 4.35	16.43 ± 5.71	*p* = 0.029

A significant negative correlation was observed between total retinal sensitivity and logMAR CDVA (***P*** **< 0.001)**, indicating that poorer visual acuity was associated with lower retinal sensitivity. Although total retinal sensitivity showed a negative trend with age, this correlation did not reach statistical significance (*P* = 0.065). No significant correlations were detected between total retinal sensitivity and axial length, intraocular pressure, choroidal flow area, FAZ metrics, or vessel densities in either the superficial or deep vascular plexus. Conversely, a significant positive correlation was found between total retinal sensitivity and the number of retinal breaks, suggesting that eyes with a greater number of breaks exhibited higher retinal sensitivity.

### Postoperative complications

Postoperative complications, including macular hole (one eye in group B), epiretinal membrane (one eye in each group), and cystoid macular oedema (four eyes in group A and two eyes in group B), were infrequent and showed no statistically significant differences between the two groups (all *P* > 0.05).

IOP elevation related to SO emulsification was not encountered in both groups. At the last follow up, the mean IOP in Group A was 12.19 ± 3.02 mmHg, while in Group B, it was 11.43 ± 2.36 mmHg, with a p-value of 0.28, indicating no significant variation between the two groups.

### Secondary interventions

Secondary surgical interventions were required in 3 eyes (8.8%) in Group A and 1 eye (3%) in Group B, with no statistically significant difference between the groups (*P* = 0.237). In Group A, all three eyes underwent reoperation to achieve retinal reattachment, whereas in Group B, one eye required surgery for a secondary full-thickness macular hole identified at the one-month postoperative follow-up (Table 2).

## Discussion

Several studies have evaluated the effects of silicone oil (SO) on the visual and anatomical outcomes of rhegmatogenous retinal detachment (RRD). However, most were retrospective and subject to selection bias, as more severe cases were preferentially treated with SO [2, 10–14]. To minimize this bias, we conducted a prospective study including only fresh (< 1 month), uncomplicated RRD with PVR grade A or B to assess outcomes after different SO tamponade durations.

Although Dubroux et al. conducted a balanced comparative study evaluating the effect of tamponade duration on silicone-oil–related retinal changes after successful RRD surgery, comparing groups with median tamponade durations of 4 (3–5) and 8 (7–9) months, the study was retrospective in nature [15]. 

 Dubroux et al. confirmed that silicone oil (SO) induces significant structural retinal changes, evidenced by increased central macular thickness (CMT) after SO removal (SOR) [15]. Shehri et al. analyzed macular and subfoveal choroidal thickness (SFCT) changes after macula-on RRD repair with SO using SD-OCT and concluded that SO-related macular thinning was temporary and reversible after SOR [12]. Similarly, two studies reported transient central retinal thinning resolving post-SOR [13, 14], whereas others found persistent retinal thinning, particularly in macula-on RRD, suggesting a lasting deleterious effect [16–19]. Zhou et al. also demonstrated a marked reduction in nerve fiber layer thickness following SO tamponade in RRD eyes [9]. Rabina et al., using a similar design to our study, found that SO caused a temporary CMT decrease, mainly affecting the inner retinal layers (IRL) [13]. Inan et al. observed thinning of the ganglion cell, outer plexiform, and outer nuclear layers in the SO group [20], while Liu et al. reported reduced parafoveal vessel density and inner retinal thickness in macula-on RRD eyes with SO [21]. Collectively, these studies indicate that SO tamponade significantly impacts the thickness of the retinal layers in RRD eyes.

The exact mechanism underlying SO-induced thinning of retinal layers, including the RNFL, remains unclear. Mechanical stress exerted by SO on the fovea may cause early loss of outer nuclear layer cell bodies [22]. Although mechanical stress may be associated with elevated IOP, all cases of IOP rise in our study were promptly managed with IOP-lowering agents, and no eyes exhibited persistent elevation. On the other hand, severe optic neuropathy may result from subretinal migration of SO [23], potentially mediated by macrophage transport of emulsified oil droplets [24]. However, no subretinal migration was observed in our series. Other proposed mechanisms include SO-related inflammatory or ionic disturbances [25], Müller cell dysfunction leading to altered potassium homeostasis and neuronal apoptosis [26], or diffusion of micromolecules from SO into the retina causing toxicity [27, 28]. Retinal dehydration due to SO’s hydrophobicity has also been suggested [13], though the presence of a retro-silicone fluid layer argues against this. Despite these hypotheses, all patients in our study received highly purified, solvent-free SO without evidence of emulsification or migration on SD-OCT. Regardless of the mechanism, Rabina et al. reported that SO-induced retinal thinning begins within four months after injection and progresses gradually thereafter [13]. 

There is a scarcity of studies examining the effect of tamponade duration on retinal thickness [13, 18, 19], and none demonstrated a significant relationship, suggesting that a tamponade period of 3–6 months may be insufficient to reveal such an effect. Consistent with these findings, our results indicate that retinal thickness changes following SOR are not influenced by tamponade duration, with no apparent differences observed between the two groups.

### Corrected distance visual acuity (CDVA)

Previous studies have reported inconsistent findings regarding the impact of silicone oil (SO) tamponade duration on visual outcomes. Some have suggested a negative effect of prolonged SO tamponade on final visual acuity (VA) [29, 30], with others observing postoperative VA decline and hypothesizing retinal toxicity associated with SO use [31, 32]. Optic disc pallor has also been reported more frequently after extended SO tamponade, possibly indicating optic nerve involvement; [33] however, we did not assess this parameter, as pallor may result from multiple factors, such as the extent of laser retinopexy. Comparisons with our findings are limited, as several studies did not account for confounding factors such as preoperative macular status. Nonetheless, our results align with those of Lee et al., who found no correlation between tamponade duration and final CDVA after adjusting for age, sex, and axial length in patients undergoing PPV with SO tamponade [19]. 

### Optical coherence tomography angiography

 Lee et al. [34] reported a significant association between the duration of silicone oil (SO) tamponade and reduced deep capillary plexus (DCP) vessel density (VD), suggesting that prolonged SO exposure may exert detrimental effects on retinal microvasculature. In contrast, Nassar et al. [35] found no significant pre- and post-SO removal differences in VD of the superficial (SCP), deep (DCP), or choriocapillaris (CCP) plexuses, nor in foveal avascular zone (FAZ) size, though a significant postoperative increase in central foveal thickness (CFT) and optic nerve VD was observed where OCTA analysis of the optic nerve demonstrated a significant postoperative increase in vessel density (VD), with whole-image VD rising from 38.39 ± 5.53% to 41.31 ± 5.97% and peripapillary capillary plexus VD increasing from 39.45 ± 7.33% to 43.04 ± 7.07% (*P* < 0.001 and *P* = 0.002, respectively). Consistent with these findings, although we did not assess vascular changes pre- and post-SO removal, our results demonstrated lower SCP and DCP VDs, as well as reduced FAZ parameters and choroidal flow area in eyes with longer SO tamponade duration, albeit without statistical significance.

Our study found no association between postoperative CDVA and either superficial or deep FAZ parameters, consistent with the findings of Sato et al. and Hong et al. [36, 37] Similarly, no correlation was observed between postoperative CDVA and SVP/DVP vessel densities, in agreement with Wang et al. [38] It is noteworthy that Sato et al., Hong et al., and Wang et al. utilized gas as the tamponading agent, in contrast to silicone oil used in our study.

Conversely, Wang et al. reported a positive correlation between CDVA and choriocapillaris flow density, highlighting the critical role of the choriocapillaris in determining final visual outcomes [38]. Similarly, our correlation analysis revealed a comparable trend—although not statistically significant—where Group A demonstrated slightly better visual outcomes (CDVA: 0.83 ± 0.26 vs. 0.91 ± 0.29) and marginally higher choroidal flow area or choriocapillaris flow density (2.05 ± 0.39 vs. 2.00 ± 0.33) compared with Group B.

 Christou et al. investigated 14 eyes that underwent a single successful PPV with silicone oil tamponade for macula-off RRD, assessing macular microcirculation using OCTA one month postoperatively. They reported a significant inverse correlation between postoperative logMAR visual acuity and foveal, parafoveal, and perifoveal vessel and perfusion densities in the superficial capillary plexus (all *P* < 0.001), while no significant correlation was found between postoperative visual acuity and FAZ parameters [39]. 

### Retinal sensitivity

Our study demonstrated no statistically significant difference in CDVA between the two groups. However, microperimetry results revealed significantly better retinal sensitivities in the outer and middle rings in Group A, with the inner ring also showing a non-significant trend toward higher sensitivity. This finding may suggest that the fovea and adjacent central retina are relatively more resilient to the potential toxic effects of silicone oil (SO). It is also plausible that a longer duration of SO tamponade might have resulted in a more pronounced and statistically significant difference. Importantly, these results highlight that visual function should not be evaluated solely based on visual acuity measures.

Consistent with our findings, Dou et al. [40] reported that 2° and 6° mean retinal sensitivities (MRS) were significantly inversely correlated with both the duration of SO tamponade (*r* = − 0.690 and − 0.764, respectively; *P* < 0.01) and the interval between retinal detachment and surgery (*r* = − 0.447 and − 0.517, respectively; *P* < 0.01), while being positively correlated with follow-up duration after SO removal (*r* = 0.290 and 0.281, respectively; *P* < 0.05). These results support the notion that retinal sensitivity, initially compromised by prolonged SO tamponade and delayed surgery, can gradually recover following SO removal.

We observed a significant positive correlation between total retinal sensitivity and the number of retinal breaks, with patients exhibiting multiple breaks demonstrating better final retinal sensitivity. Upon reviewing the clinical data, it was noted that 3 of 6 patients in Group A and 3 of 4 patients in Group B had more than one retinal break. This finding may be explained by the fact that patients with multiple breaks likely experienced a more acute onset of visual symptoms, possibly due to a more pronounced posterior vitreous detachment (PVD) process accompanied by vitreous hemorrhage or rapid retinal detachment progression. Consequently, these patients may have sought medical attention earlier, leading to more timely surgical intervention and preservation of retinal function.

### Postoperative complications

#### Silicone oil-related visual loss (SORVL)

In our study, there were no significant differences between the two groups regarding intraoperative or postoperative complications. Notably, no cases of silicone oil–related visual loss (SORVL) were observed in either study arm.

In contrast, Scheerlinck et al., across three separate studies, reported cases of SORVL and demonstrated that its occurrence appeared independent of both the surgeon and the surgical technique employed [41–43]. SORVL—defined as a loss of more than two Snellen lines during or following silicone oil tamponade without any other identifiable cause—has been described predominantly in cases involving giant retinal tears and macula-sparing retinal detachments, with reported incidences of up to 50% [10]. 

The proposed mechanisms underlying SORVL include prolonged tamponade duration, altered retinal metabolism, increased concentrations of fibrogenic growth factors within the retro-oil fluid, phototoxic damage from ultraviolet exposure, and depletion of key antioxidants and lipid-soluble factors such as lutein, zeaxanthin, and ascorbate from the vitreous humour [6]. Clinically, SORVL may manifest as inner retinal thinning, scotomas, visual field defects, or diminished electrophysiological responses, with some studies suggesting associated microcirculatory impairment [6]. 

#### Intraocular pressure

To our knowledge, few studies have compared intraocular pressure (IOP) changes between early and delayed silicone oil removal (SOR). Jonas et al. conducted a study involving 198 patients who underwent pars plana vitrectomy (PPV) with 5,000-centistoke silicone oil endotamponade and were followed for at least three months after oil removal. They reported that IOP was statistically independent of the duration of silicone oil tamponade (*P* > 0.20) [44]. Consistent with their findings, our study demonstrated no significant difference in IOP between the early and delayed SOR groups.

Interestingly, Sharma et al. found a positive correlation between IOP on the first postoperative day and final (6 months) logMAR CDVA (*P* = 0.04, r = + 0.24) [45]. In contrast, our correlation analysis did not reveal such an association, which may be attributed to our rigorous postoperative monitoring protocol and prompt initiation of medical management for any IOP elevation within hours of PPV and silicone oil injection.

This study incorporated several methodological strengths and acknowledged limitations. To minimize the influence of surgical factors, intraoperative intraocular pressure (IOP) was standardized at 25 mmHg using the Alcon Constellation system, and complex procedures such as internal limiting membrane (ILM) peeling, proliferative membrane removal, and retinectomies were excluded. Phacoemulsification was uniformly performed prior to PPV or silicone oil (SO) removal, and all outcome assessments were conducted at least one-month post–SO removal to allow for stabilization of postoperative inflammation and IOP fluctuations. All pars plana vitrectomy surgeries were performed by the same surgeon and all silicone oil removal surgeries were performed by another but same surgeon. These measures helped standardize the procedure and isolate the effects attributable specifically to SO on retinal sensitivity and microcirculation.

However, certain limitations should be noted. The single-center design and relatively small sample size may have limited the statistical power to detect subtle differences between study groups. Notably, re-detachment occurred only in Group A only and although the difference was not statistically significant, the absence of re-detachment in Group B should not be overlooked. The possibility that the shorter SO tamponade duration in Group A resulted in insufficient tamponade should be further studied with larger sample size. The follow-up period was also relatively short, potentially underestimating long-term anatomical and visual changes—particularly in cases with prolonged SO retention beyond six months. Additionally, although all patients had clear ocular media at one-month post-SO removal, subtle optical aberrations may have influenced retinal sensitivity and CDVA measurements; the use of modern aberrometers could have provided a more precise assessment.

In conclusion: Although the differences did not reach statistical significance, both structural and functional outcomes tended to be better with early silicone oil (SO) removal, with no observed increase in postoperative complication rates. Microperimetry and macular sensitivity results were significantly superior in the early removal group (Group A) compared to the delayed removal group (Group B), suggesting improved visual function with earlier SO removal. These findings highlight that postoperative visual function following pars plana vitrectomy (PPV) should not be assessed solely by corrected distance visual acuity (CDVA); incorporating microperimetry provides a more comprehensive evaluation of macular function and visual performance.

## Data Availability

All data generated or analysed during this study are included in this published article.

## References

[1] Warren A, Wang DW, Lim JI. Rhegmatogenous retinal detachment surgery: A review. Clin Exp Ophthalmol. 2023;51(3):271–9. 10.1111/ceo.14205. 36640144 10.1111/ceo.14205 PMC12489960

[2] Kounatidou NE, Mautone L, Druchkiv V, Spitzer MS, Skevas C. Early versus late silicone oil tamponade removal after rhegmatogenous retinal detachment: a retrospective real world comparative study. Int J Retina Vitreous. 2025;11(1):105. 10.1186/s40942-025-00743-9. 41084092 10.1186/s40942-025-00743-9 PMC12516905

[3] Chen ZY, Wu YQ, Liu BY, Ma Y, Lin ZL, Duan RP, et al. Short-term silicone oil tamponade on retinal structure and function in rhegmatogenous retinal detachment: a randomized controlled trial. Int J Ophthalmol. 2026;19(1):83–9. 10.18240/ijo.2026.01.11. eCollection 2026. 41524006 10.18240/ijo.2026.01.11 PMC12782080

[4] Gisquet C, Ndiaye NC, Dubroux C, Angioi-Duprez K, Berrod JP, Conart JB. Retinal redetachment after silicone oil removal: a risk factor analysis. BMC Ophthalmol. 2024;24(1):346. 10.1186/s12886-024-03618-z. 10.1186/s12886-024-03618-z PMC11325823 39148018

[5] Chen Y, Kearns VR, Zhou L, Sandinha T, Lam WC, Steel DH, et al. Silicone oil in vitreoretinal surgery: indications, complications, new developments and alternative long-term tamponade agents. Acta Ophthalmol. 2021;99(3):240–50. 10.1111/aos.14604. 32930501 10.1111/aos.14604

[6] Scheerlinck LM, Schellekens PA, Liem AT, Steijns D, Leeuwen R, Incidence. Risk Factors, and clinical characteristics of unexplained visual loss after intraocular silicone oil for Macula-On retinal detachment. Retina. 2016;36(2):342–50. 10.1097/IAE.0000000000000711. 26308530 10.1097/IAE.0000000000000711

[7] Zhang Z, Zhang X, Yao T, Chen J, Huang L, Qiu C, et al. Effect of long-term silicone oil tamponade on the density of blood vessels in the macular and peripapillary region in patients with rhegmatogenous retinal detachment. Int Ophthalmol. 2025;45(1):1–0. 10.1007/s10792-025-03460-2. 10.1007/s10792-025-03460-2 40131517

[8] Machemer R, Aaberg TM, Freeman HM, Irvine AR, Lean JS, Michels RM. An updated classification of retinal detachment with proliferative vitreoretinopathy. Am J Ophthalmol. 1991;15(2):159–65. 10.1016/s0002-9394(14)76695-4. 10.1016/s0002-9394(14)76695-4 1867299

[9] Zhou Y, Zhang S, Zhou H, Gao M, Liu H, Sun X. Comparison of fundus changes following silicone oil and sterilized air tamponade for macular-on retinal detachment patients. BMC Ophthalmol. 2020;20(1):249. 10.1186/s12886-020-01523-9. 32571251 10.1186/s12886-020-01523-9 PMC7310510

[10] Park W, Kim M, Kim RY, Kim JY, Kwak JH, Park Y-G, et al. Long-term visual prognosis and characteristics of recurrent retinal detachment after silicone oil removal. PLoS ONE. 2023;18(2):e0265162. 10.1371/journal.pone.0265162. 36753472 10.1371/journal.pone.0265162 PMC9907833

[11] Ferrara M, Coco G, Sorrentino T, Jasani KM, Moussa G, Morescalchi F, et al. Retinal and corneal changes associated with intraocular silicone oil tamponade. J Clin Med. 2022;11(17):5234–37. 10.3390/jcm11175234. 36079165 10.3390/jcm11175234 PMC9457190

[12] Al-Shehri AM, Aljohani S, Aldihan KA, Alrashedi MJ, Alrasheed S, Schatz P. Effect of silicone oil versus gas tamponade on macular layer microstructure after Pars plana vitrectomy for macula on rhegmatogenous retinal detachment. BMC Ophthalmol. 2024;24(1):119. 10.1186/s12886-024-03377-x. 38486220 10.1186/s12886-024-03377-x PMC10938769

[13] Rabina G, Azem N, Barequet D, Barak A, Loewenstein A, Schwartz S. Silicone oil tamponade effect on macular layer thickness and visual acuity. Retina. 2020;40(5):998–1004. 10.1097/IAE.0000000000002464. 30707147 10.1097/IAE.0000000000002464

[14] Lo DM, Flaxel CJ, Fawzi AA. Macular effects of silicone oil tamponade: optical coherence tomography findings during and after silicone oil removal. Curr Eye Res. 2017;42(1):98–103. 10.3109/02713683.2016.1146776. 10.3109/02713683.2016.1146776 27409721

[15] Dubroux C, Salleron J, Angioi-Duprez K, Berrod J-P, Conart J-B. Effect of duration of silicone oil tamponade on retinal structure after rhegmatogenous retinal detachment surgery. Ophthalmologica. 2022;245(2):144–51. 10.1159/000519520. 34929691 10.1159/000519520

[16] Christensen UC, la Cour M. Visual loss after use of intraocular silicone oil associated with thinning of inner retinal layers. Acta Ophthalmol. 2012;90(8):733–7. 10.1111/j.1755-3768.2011.02248.x. 21914150 10.1111/j.1755-3768.2011.02248.x

[17] Caramoy A, Droege KM, Kirchhof B, Fauser S. Retinal layers measurements in healthy eyes and in eyes receiving silicone oil-based endotamponade. Acta Ophthalmol. 2014;92(4):e292–7. 10.1111/aos.12307. 24238324 10.1111/aos.12307 PMC4153956

[18] Purtskhvanidze K, Hillenkamp J, Tode J, Junge O, Hedderich J, Roider J, et al. Thinning of inner retinal layers after vitrectomy with silicone oil versus gas endotamponade in eyes with macula-off retinal detachment. Ophthalmologica. 2017;238(3):124–32. 10.1159/000477743. 28719903 10.1159/000477743

[19] Lee SH, Han JW, Byeon SH, Kim SS, Koh HJ, Lee SC, et al. Retinal layer segmentation after silicone oil or gas tamponade for macula-on retinal detachment using optical coherence tomography. Retina. 2018;38(2):310–9. 10.1097/IAE.0000000000001533. 28207606 10.1097/IAE.0000000000001533

[20] Inan S, Polat O, Ozcan S, Inan UU. Comparison of long-term automated retinal layer segmentation analysis of the macula between silicone oil and gas tamponade after vitrectomy for rhegmatogenous retinal detachment. Ophthalmic Res. 2020;63(6):524–32. 10.1159/00050638226. 32036367 10.1159/000506382

[21] Liu Y, Lei B, Jiang R, Huang X, Zhou M, Xu G. Changes of macular vessel density and thickness in gas and silicone oil tamponades after vitrectomy for macula-on rhegmatogenous retinal detachment. BMC Ophthalmol. 2021;21(1):392. 10.1186/s12886-021-02160-627. 34781932 10.1186/s12886-021-02160-6 PMC8591799

[22] Dooley I, Treacy M, O’Rourke M, Khaild I, Kilmartin D. Serial spectral domain ocular coherence tomography measurement of outer nuclear layer thickness in rhegmatogenous retinal detachment repair. Curr Eye Res. 2015;40(10):1073–6. 10.3109/02713683.2014.97193628. 25328979 10.3109/02713683.2014.971936

[23] Majid M, Hussin H, Biswas S, Haynes R, Mayer E, Dick A. Emulsification of Densiron-68 used in inferior retinal detachment surgery. Eye. 2008;22(1):152–7. 10.1038/sj.eye.670278429. 17401320 10.1038/sj.eye.6702784

[24] Budde M, Cursiefen C, Holbach LM, Naumann GO. Silicone oil–associated optic nerve degeneration. Am J Ophthalmol. 2001;131(3):392–4. 10.1016/s0002-9394(00)00800-x. 11239883 10.1016/s0002-9394(00)00800-x

[25] Winter M, Eberhardt W, Scholz C, Reichenbach A. Failure of potassium siphoning by Muller cells: a new hypothesis of perfluorocarbon liquid–induced retinopathy. Investig Ophthalmol Vis Sci. 2000;41(1):256–61. PMID: 10634628. 10634628

[26] Newsom RS, Johnston R, Sullivan PM, Aylward GB, Holder GE, Gregor ZJ. Sudden visual loss after removal of silicone oil. Retina. 2004;24(6):871–7. 10.1097/00006982-200412000-00005. 10.1097/00006982-200412000-00005 15579983

[27] Gonvers M, Hornung J-P, de Courten C. The effect of liquid silicone on the rabbit retina: histologic and ultrastructural study. Arch Ophthalmol. 1986;104(7):1057–62. 10.1001/archopht.1986.01050190115049. 3729775 10.1001/archopht.1986.01050190115049

[28] Gabel V-P, Kampik A, Burkhardt J. Analysis of intraocularly applied silicone oils of various origins. Graefes Arch Clin Exp Ophthalmol. 1987;225(3):160–2. 10.1007/BF02175441. 3609755 10.1007/BF02175441

[29] Durrani AK, Rahimy E, Hsu J. Outer retinal changes on spectral-domain optical coherence tomography pre-and post-silicone oil removal. Ophthalmic Surg Lasers Imaging Retina. 2017;48(12):978–82. 10.3928/23258160-20171130-04. 29253300 10.3928/23258160-20171130-04

[30] Eibenberger K, Sacu S, Rezar-Dreindl S, Schmidt-Erfurth U, Georgopoulos M. Silicone oil tamponade in rhegmatogenous retinal detachment: functional and morphological results. Curr Eye Res. 2020;45(1):38–45. 10.1080/02713683.2019.1652917. 31478404 10.1080/02713683.2019.1652917

[31] Yorston D, Wickham L, Benson S, Bunce C, Sheard R, Charteris D. Predictive clinical features and outcomes of vitrectomy for proliferative diabetic retinopathy. Br J Ophthalmol. 2008;92(3):365–8. 10.1136/bjo.2007.124495. 18303158 10.1136/bjo.2007.124495

[32] Jackson TL, Johnston RL, Donachie PH, Williamson TH, Sparrow JM, Steel DH. The Royal college of ophthalmologists’ National ophthalmology database study of vitreoretinal surgery: report 6, diabetic vitrectomy. JAMA Ophthalmol. 2016;134(1):79–85. 10.1001/jamaophthalmol.2015.4587. 26584210 10.1001/jamaophthalmol.2015.4587

[33] Shroff CM, Gupta C, Shroff D, Atri N, Gupta P, Dutta R. Bimanual microincision vitreous surgery for severe proliferative diabetic retinopathy: outcome in more than 300 eyes. Retina. 2018;38:S134–45. 10.1097/IAE.0000000000002093. 29406470 10.1097/IAE.0000000000002093

[34] Lee J, Cho H, Kang M, Hong R, Seong M, Shin Y. Retinal changes before and after silicone oil removal in eyes with rhegmatogenous retinal detachment using swept-source optical coherence tomography. J Clin Med. 2021;10(22):5436. 10.3390/jcm10225436. 34830717 10.3390/jcm10225436 PMC8619201

[35] Nassar GA, Makled HS, Youssef MM, Hassan LM. Functional and perfusion changes associated with silicone oil tamponade after macula-off rhegmatogenous retinal detachment surgery: an optical coherence tomography angiography/microperimetry study. Int Ophthalmol. 2024;44(1):107. 10.1007/s10792-024-03037-5. 38386180 10.1007/s10792-024-03037-5 PMC10884141

[36] Sato T, Kanai M, Busch C, Wakabayashi T. Foveal avascular zone area after macula-off rhegmatogenous retinal detachment repair: an optical coherence tomography angiography study. Graefes Arch Clin Exp Ophthalmol. 2017;255(10):2071–2. 10.1007/s00417-017-3743-5. 28736790 10.1007/s00417-017-3743-5

[37] Hong EH, Cho H, Kang MH, Shin YU, Seong M. Changes in retinal vessel and retinal layer thickness after vitrectomy in retinal detachment via swept-source OCT angiography. Invest Ophthalmol Vis Sci. 2020;61(2):35. 10.1167/iovs.61.2.35. 32084264 10.1167/iovs.61.2.35 PMC7326598

[38] Wang H, Xu X, Sun X, Ma Y, Sun T. Macular perfusion changes assessed with optical coherence tomography angiography after vitrectomy for rhegmatogenous retinal detachment. Graefes Arch Clin Exp Ophthalmol. 2019;257(4):733–40. 10.1007/s00417-019-04273-7. 30796563 10.1007/s00417-019-04273-7

[39] Christou EE, Stavrakas P, Georgalas I, Batsos G, Christodoulou E, Stefaniotou M. Macular microcirculation changes after macula-off rhegmatogenous retinal detachment repair with silicone oil tamponade evaluated by OCT-A: preliminary results. Ther Adv Ophthalmol. 2022 Jun:14:25158414221105222. 10.1177/25158414221105222. 10.1177/25158414221105222 PMC9208039 35734223

[40] Dou R, Li R, Li R-c, Yu Y-r, Zhou J-x, Li R-m, et al. Evaluation of retinal structural and functional changes after silicone oil removal in patients with rhegmatogenous retinal detachment: a retrospective study. Int J Retina Vitreous. 2024;10(1):1. 10.1186/s40942-023-00519-z. 38167553 10.1186/s40942-023-00519-z PMC10759386

[41] Scheerlinck LM, Schellekens PA, Liem AT, Steijns D, van Leeuwen R. Retinal sensitivity following intraocular silicone oil and gas tamponade for rhegmatogenous retinal detachment. Acta Ophthalmol. 2018;96(6):641–7. 10.1111/aos.13685. 29498239 10.1111/aos.13685

[42] Scheerlinck LM, Kuiper JJ, Liem AT, Schellekens PA, van Leeuwen R. Electrolyte composition of retro-oil fluid and silicone oil‐related visual loss. Acta Ophthalmol. 2016;94(5):449–53. 10.1111/aos.12959. 26806559 10.1111/aos.12959

[43] Scheerlinck LM, Schellekens PA, Liem AT, Steijns D, Van Leeuwen R. Incidence, risk factors, and clinical characteristics of unexplained visual loss after intraocular silicone oil for macula-on retinal detachment. Retina. 2016;36(2):342–50. 10.1097/IAE.0000000000000711. 26308530 10.1097/IAE.0000000000000711

[44] Jonas JB, Knorr HL, Rank RM, Budde WM. Intraocular pressure and silicone oil endotamponade. J Glaucoma. 2001;10(2):102–8. 10.1097/00061198-200104000-00006. 11316091 10.1097/00061198-200104000-00006

[45] Sharma YR, Pruthi A, Azad RV, Kumar A, Mannan R. Impact of early rise of intraocular pressure on visual outcome following diabetic vitrectomy. Indian J Ophthalmol. 2011;59(1):37–40. 10.4103/0301-4738.73724. 21157070 10.4103/0301-4738.73724 PMC3032240

